# Cuprorivaite microspheres inhibit cuproptosis and oxidative stress in osteoarthritis via Wnt/β-catenin pathway

**DOI:** 10.1016/j.mtbio.2024.101300

**Published:** 2024-10-16

**Authors:** Bo Li, Tongmeng Jiang, Juan Wang, Hongping Ge, Yaqi Zhang, Tong Li, Chen Wang, Weiguo Wang

**Affiliations:** a Department of Orthopedics, China-Japan Friendship Hospital, Beijing 100029, China; b Key Laboratory of Emergency and Trauma of Ministry of Education, Key Laboratory of Haikou Trauma, Key Laboratory of Hainan Trauma and Disaster Rescue, The First Affiliated Hospital, Hainan Medical University, Haikou 571199, China; c Engineering Research Center for Hainan Bio-Smart Materials and Bio-Medical Devices, College of Emergency and Trauma, Hainan Academy of Medical Sciences, Hainan Medical University, Haikou 571199, China; d Key Laboratory of Tropical Translational Medicine of Ministry of Education & Key Laboratory of Brain Science Research and Transformation in Tropical Environment of Hainan Province, Hainan Provincial Stem Cell Research Institute, School of Basic Medicine and Life Sciences, Hainan Medical University, Haikou, 571199, China; e Wenzhou Institute, University of Chinese Academy of Sciences, Wenzhou 325000, China; f Department of Dermatology, The Second Affiliated Hospital and Yuying Children's Hospital of Wenzhou Medical University, Wenzhou 325000, China

**Keywords:** Osteoarthritis, Cuprorivaite microspheres, Cuproptosis, Oxidative stress, Wnt/β-catenin pathway

## Abstract

This study aims to evaluate the therapeutic potential of cuprorivaite microspheres for osteoarthritis (OA), in particular, potential molecular mechanisms were investigated. The microspheres were developed from Ca(NO_3_)_2_•4H_2_O, Cu(NO_3_)_2_•3H_2_O, and silica gel, and further therapeutic effects were tested *in vitro* on mouse primary chondrocytes treated with interleukin-1β (IL-1β) to mimic OA, and *in vivo* on OA mice induced via anterior cruciate ligament transection (ACLT) surgery. The microspheres were shown to mitigate IL-1β-induced apoptotic, inflammatory, oxidative stress and cuproptosis markers while enhancing cell viability and extracellular matrix (ECM) components in chondrocytes. Moreover, the microspheres ameliorated histopathological damage, reduced inflammatory, oxidative stress and cuproptosis markers, and enhanced ECM biomarker levels in OA mice, implicating their role in suppressing cuproptosis and oxidative stress. The aforementioned effects of the cuprorivaite microspheres were demonstrated by using SKL2001, an agonist of the Wnt/β-catenin pathway. The results suggest cuprorivaite microspheres as a promising intervention for OA and cartilage regeneration, highlighting their therapeutic effects on cellular and molecular levels.

## Graphical abstract

**Figure:**
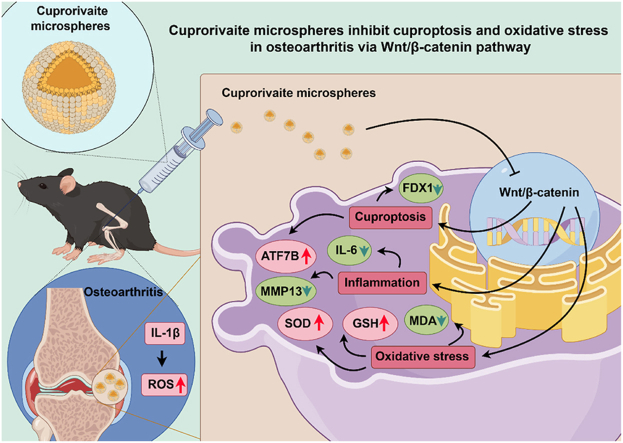

## 1 Introduction

Osteoarthritis (OA) is a common, degenerative joint disease characterized by joint pain and stiffness in clinics. In OA, extracellular matrix (ECM) degradation in chondrocytes is the main cause of OA pathology, with degradation of ECM components such as collagen II and proteoglycans induced by matrix metalloproteinase 13, which further contributes to the death and inflammation in chondrocytes [1]. Conventional treatments for OA include surgery and pharmacotherapy. However, the evidence supporting surgical interventions requires further substantiation, oral medications offer only modest benefits, and the majority of these therapies do not outperform placebo [2]. There is still a lack of effective drugs to reverse or terminate OA progression. Therefore, the development of effective OA drugs remains a focus of current studies.

Research indicates that during the progression of OA, there is an elevated apoptosis level in osteoblasts, leading to an imbalance between bone anabolism and catabolism [3]. This imbalance causes a loss of the original stress-supporting capacity of the subchondral bone and promotes the formation of osteophytes, ultimately resulting in the deterioration of the joint structure [4]. Copper metabolism is precisely regulated within cells [5], and it notably impacts cartilage and bone engineering [6]. In normal conditions, Cu^2+^ is reduced to Cu^+^, translocating into the intracellular environment for mitochondrial respiration control or gene expression modulation in the nucleus [5]. ATPase copper transporter 7 B (ATP7B) performs the extracellular release of Cu^+^ [7]. However, deregulation in the mechanism, in the pathological condition, of copper metabolism leads to a novel type of cell death, named cuproptosis [8]. In this process, large amounts of Cu^2+^ enter the cells directly to bind to dihydrolipoamide S-acetyltransferase (DLAT), thereby causing the heteropolymerisation of DLAT that induces cytoxicity and death in cells [9]. Meanwhile, ferredoxin 1 (FDX1) catalyzes Cu^2+^ reduced to toxic Cu ^+^ that evokes the degradation of Fe-S cluster and proteotoxic reactions; it is also capable of inducing mitochondrial dysfunction and oxidative stress via mediating the sulfooctanoylation of DLAT and mitochondrial proteins, which finally results in the death in cells [10]. Copper metabolism impairment and cuproptosis are related to the dysfunction in bone hemostasis. There is an intriguing insight that cuproptosis may function as the risk factor for OA pathogenesis [11]. For example, Wang et al. observed the elevation of FDX1 in patients with OA as compared to the control group [12]. DLAT in patients with OA is highly expressed and shows a relationship to Th17-mediated immune responses [13]. Thus, cuproptosis activation is a common phenomenon in OA. Nevertheless, the cuproptosis-related mechanism in OA remains unclear, including initiation, propagation and execution.

Copper-doped bioactive microspheres are capable of promoting bone formation and inhibiting bone loss since their advances in pore distribution and mechanical properties of biomaterials that elevate cell attachment and bone formation [14]. Moreover, copper can enhance the properties of materials via suppressing bacterial biofilm formation [15]. Egyptian blue (CaCuSi_4_O_10_), the major component of the cuprorivaite microspheres, has the huge potential to promote osteogenesis. He et al. constructed a biomaterial based on CaCuSi_4_O_10_ and investigated its osteogenic capability *in vitro* and *in vivo* [16]. They found that 3D-printing CaCuSi_4_O_10_-based scaffold promoted cell attachment and then elevated bone formation. Since subchondral bone loss is a major factor in the progression of OA, inhibiting it may help alleviate the condition [17]. Unfortunately, it remains unclear whether cuprorivaite microspheres can alleviate OA, despite their demonstrated therapeutic effects on bone loss. Additionally, the potential mechanisms by which they might exert these effects are not yet understood.

The Wnt/β-catenin pathway comprises a range of proteins such as Wnt1, glycogen synthase kinase 3β (GSK3β), and β-catenin. Without Wnt signaling, β-catenin in the cytoplasm is captured by a ‘cleavage complex’ containing GSK3β, followed by proteasome-mediated degradation. The activated Wnt1 interrupts the binding between β-catenin and the complex, thereby promoting β-catenin shift to the nucleus in which β-catenin modulates transcription activation of target genes [18]. The Wnt/β-catenin pathway has the major role of maintaining cartilage homeostasis. According to previous reports, β-catenin expressed in cartilaginous tissue is positively related to the severity of OA [19,20]. In adult mice, Wnt/β-catenin pathway activation causes the OA-like phenotype, with cartilage loss and abnormally expressed biomarkers in chondrocytes [21]. As shown in a mouse model of OA, inhibition of the Wnt/β-catenin pathway can inhibit local inflammation and cartilage loss [22]. The senescence of chondrocytes is a crucial mechanism in the pathogenesis of OA, and it is associated with the Wnt/β-catenin pathway. For instance, a previous study demonstrated that the Wnt/β-catenin pathway promotes the senescence phenotype of chondrocytes through the downregulation of SIRT1 and the acetylation of p53 [23]. From this, it can be inferred that targeting the Wnt/β-catenin pathway may represent a promising therapeutic strategy for the treatment of OA. Wnt/β-catenin pathway and cuproptosis are of particular significance for the occurrence and development of OA. Thus, it is also worth discussing whether cuprorivaite microspheres are capable of regulating cuproptosis and Wnt/β-catenin pathway activation during its treatment for OA.

Here, we integrated Ca(NO_3_)_2_•4H_2_O, Cu(NO_3_)_2_•3H_2_O and silica gel to develop cuprorivaite microspheres in the present study. These microspheres were used to treat the inflammatory chondrocyte and mice OA model. In this way, we aimed to demonstrate the therapeutic role of cuprorivaite microspheres in OA and investigate its potential capability to modulate cuproptosis via targeting Wnt/β-catenin pathway.

## 2 Methods

### 2.1 Construction of cuprorivaite microspheres

**Fig. 1:**
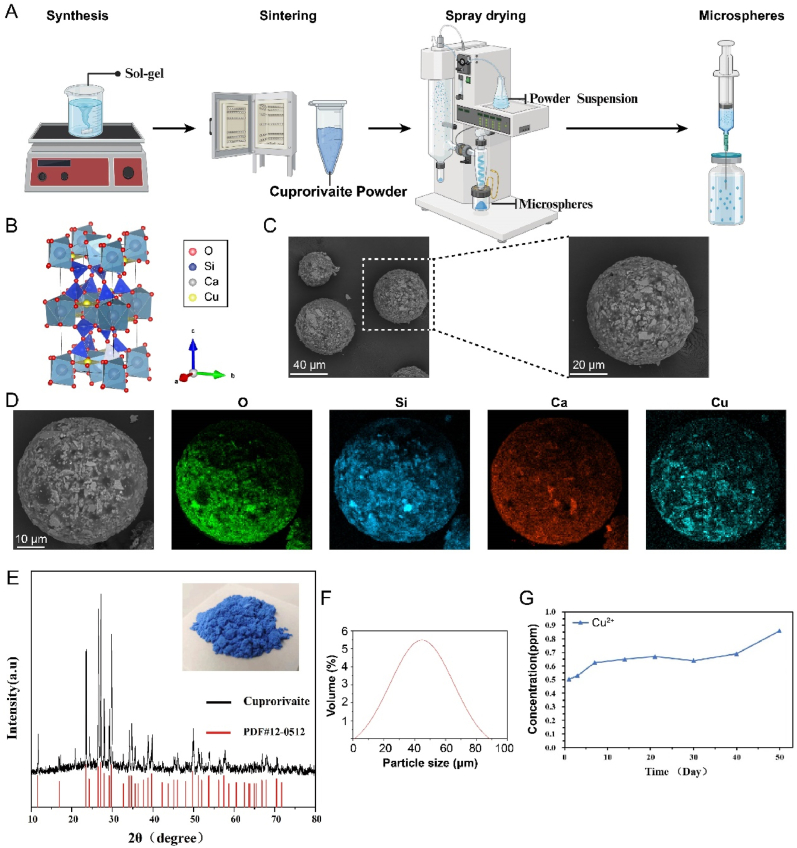
Characterization of Cuprorivaite (Egyptian blue, CaCuSi_4_O_10_) microspheres. (A) Flowchart of CaCuSi_4_O_10_ microspheres synthesis. (B) The schematic crystal structure of CaCuSi_4_O_10_ using the spatial model. (C) Representative SEM images for CaCuSi_4_O_10_. (D) EDS mapping. (E) XRD examination and a representative gross image of CaCuSi_4_O_10_. (F) Particle size distributions of the microspheres. (G) Cu^2+^ release detected by inductively-coupled plasma emission spectrometer.

The synthesis process is shown in Fig. 1A. Briefly, the Egyptian blue (CaCuSi_4_O_10_, cuprorivaite) sample was synthesized using a sol-gel method. Initially, 4.83 g of copper nitrate trihydrate (Cu(NO_3_)_2_·3H_2_O) and 4.72 g of calcium nitrate tetrahydrate (Ca(NO_3_)_2_·4H_2_O) were weighed into a 100 mL beaker, followed by the addition of 4.68 mL of deionized water and 11.67 mL of absolute ethanol. The beaker was then placed in a water bath set to 40 °C and stirred at 600 rpm for 30 min to obtain a mixed solution. Subsequently, 17.81 mL of tetraethyl orthosilicate (TEOS) was slowly added while stirring, and 4 mL of 1 M HCl was used to adjust the pH to 2. The temperature of the water bath was then increased to 50 °C, and the solution was stirred at 500–600 rpm for 4 h to form a uniform gel. This gel was aged in a blast oven at 60 °C for 48 h and then transferred to another oven at 100 °C for an additional 48 h to form a xerogel. The xerogel was ground using a ball mill and passed through a 200-mesh sieve to obtain a fine powder, which was subsequently calcined in a muffle furnace at 1000 °C for 4 h. After cooling to room temperature, the sample was ground again and sieved through a 200-mesh sieve to obtain the final Egyptian blue powder. For the preparation of Egyptian blue spheres, the synthesized CaCuSi_4_O_10_ powder was dispersed in a 2 wt % PVA solution and ball-milled for 4 h using 5 mm diameter zirconia balls. The suspension was then diluted to a 30 wt % solid content and subjected to spray-drying with an inlet temperature of 280 °C, an outlet temperature of approximately 100 °C, an air pressure of 1 bar, and a feed rate of 30 mL/min. The obtained microspheres continue to be calcined at 1000 °C for 2 h to obtain the final microsphere samples used in this experiment.

Micro-morphological scanning and performance parameter characterization were conducted using scanning electron microscopy (SEM) to analyze particles that were either spherical or non-spherical. X-ray diffraction (XRD) was employed to determine the phase composition and crystallinity of the microspheres. Elemental analysis was carried out using energy-dispersive X-ray spectroscopy (EDS) for both qualitative and semi-quantitative assessments. The size distribution of the microspheres was tested using a laser diffraction particle size distribution analyzer. Additionally, the copper ion release from cuprorivaite microspheres over a 50-day period was examined using inductively coupled plasma optical emission spectroscopy (ICP-OES).

### 2.2 Animals

The animal experiments were approved by the Animal Ethics Committee (No. zryhyy21-22-08-03). C57BL/6 male mice (Slaccas, Shanghai, China; aged 6–8 weeks) were used in the present study. Mice could freely access food and water at 21 ± 1 °C with a humidity of 60 ± 1 %. A mouse model of OA was constructed using anterior cruciate ligament transection (ACLT) surgery as previously [24,25]. Mice were divided into four groups: sham, 1/16, OA and OA+1/16 group (n = 6/per group), in which the sham group received Stroke-physiological Saline Solution injection, 1/16 group received cuprorivaite microspheres with a dilution at 1/16 of the mother extraction solution, OA group underwent ACLT surgery and OA+1/16 group underwent OA modeling and cuprorivaite microspheres treatment. Cuprorivaite microspheres were treated every 3 days for 8 weeks via intra-articular injection with a dilution at 1/16 of the mother extraction solution. Since directly measuring the quality of microspheres in joint cavity tissue presents technical challenges, we employed an indirect method to theoretically calculate the mass of microspheres injected. The process is as follows: First, we determined the optimal release amount of copper ions through *in vitro* cell experiments, using a 1/16 dilution. Based on the copper ion concentration detected in this dilution by ICP (10.3259 mg/L), we calculated the theoretical amount of CaCuSi_4_O_10_ microspheres required (60.66 mg/kg) and used this as a reference dosage for animal experiments. All tests were performed at 8 weeks of postoperation.

### 2.3 Isolation and culture of mouse primary chondrocytes

Normal mice were euthanized by the dislocation method. Cartilage tissues from mouse knee joints were isolated for primary cell extraction. The mouse cartilage tissue was washed with sterile phosphate-buffered saline containing 5 × penicillin and 5 × streptomycin sulfate. Subsequently, the tissue was cut into small pieces and digested with 0.06 % type II collagenase (BioFroxx, Einhausen, Germany) for 2 h. The resulting chondrocytes were then collected and cultured in Dulbecco's Modified Eagle's Medium (DMEM)/F12 (1:1) (Gibco, USA) supplemented with 10 % fetal bovine serum (Gibco, USA).

### 2.4 Cell experiment procedures

To mimic the inflammatory chondrocytes in OA, 10 ng/mL IL-1β recombinant protein (PHC0811, Gibco, USA) was used to incubate cells for 24 h at 37 °C [26]. cuprorivaite microspheres with different dilutions of the aforementioned mother extract solution at 1/2, 1/4, 1/8, 1/16, 1/32, 1/64 and 1/128 were added to treat cells for 24 h after 24 h IL-1β stimulation. Moreover, IL-1β-stimulated chondrocytes were incubated with 40 μmol/L SKL2001 (HY-101085, MedChemExpress, USA), an agonist of Wnt/β-catenin pathway, for 15 h at 37 °C to reverse the role of IL-1β in Wnt/β-catenin pathway.

### 2.5 Cell counting kit-8 (CCK-8) for cell viability measurement

Cell viability was detected by CCK-8 kit (C0037, Beyotime, Shanghai, China). Cells seeded into 96-well plates were incubated with 10 μL CCK-8 reagent at 37 °C for 1 h. The value of optical density (OD) of cell samples was read by a multi-functional microplate detector (BioTek Synergy HTX, Agilent, USA) at 450 nm. Cell viability (%) =(OD_test_-OD_blank_)/(OD_control_-OD_blank_) × 100 %.

### 2.6 Cell viability detection

To detect cell viability, a fluorescein diacetate kit (40720ES03, Yeasen, Shanghai, China) and Propidium iodide (PI) Staining Solution (40710ES03, Yeasen) were used. Cells responded in 500 μL staining working buffer for 30 min at 37 °C, protected from light. After being washed with Hank's Buffer with Hepes (HHBS), cells were responded to in 500 μL HHBS buffer and detected by a fluorescence microscope (Olympus, Japan).

### 2.7 Cell apoptosis detection

Annexin V-FITC Apoptosis Detection Kit (C1062S, Beyotime, Shanghai, China) was used to measure cell apoptosis. Briefly, a total of 60000 cells were collected for incubation with 195 μL Annexin V-FITC binding buffer, 5 μL Annexin V-FITC and 10 μL PI for 20 min at room temperature, protected from light, followed by the detection of flow cytometry (Beckman, USA).

### 2.8 Pro-inflammatory factor detection

To detect the levels of tumor necrosis factor α (TNF-α), interleukin-6 (IL-6), matrix metalloproteinase 13 (MMP13) in a cell model of OA, cell medium was collected and centrifugated at 700 g for 10 min at 4 °C. The supernatant was centrifugated at 9, 000 g for 15 min at 4 °C again. To detect the levels of TNF-α, IL-6, and MMP13 in a mouse model of OA, serum samples were collected from mice using cardiac blood collection. Then, levels of TNF-α, IL-6, and MMP13 in cell supernatant and serum samples were detected by the multi-functional microplate detector, with mouse TNF-α (CSB-E04741m, CUSABIO, Wuhan, China), mouse IL-6 (CSB-E04639m, CUSABIO, Wuhan, China) and mouse MMP13 (CSB-E07413m, CUSABIO, Wuhan, China) kit.

### 2.9 Blood routine and biochemical measurement

Blood samples from cardiac blood collection were used for blood routine measurement using an automatic blood analyzer (BC-5000Vet; Mindray, Shenzhen, China). Also, serum samples were performed for the biochemical test of the automatic biochemical analyzer (BS-430; Mindray, Shenzhen, China), with the kits including aspartate transaminase (AST) assay kit (MAK467, Sigma-Aldrich, USA) and alanine transaminase (ALT) assay kit (MAK052, Sigma-Aldrich, USA).

### 2.10 Measurement of malondialdehyde (MDA), glutathione (GSH), superoxide dismutase (SOD), reactive oxygen species (ROS)

Chondrocyte cell supernatant and cartilage tissue homogenate were centrifugated with 10, 000 g at 4 °C for 10 min. The supernatant was used for the detection of MDA, GSH and SOD through Lipid Peroxidation MDA Assay Kit (S0131S, Beyotime, Shanghai, China), GSH and GSSG Assay Kit (S0053, Beyotime, Shanghai, China) and SOD (S0101S, Beyotime, Shanghai, China) according to the product guidelines. To characterize ROS generation in chondrocytes, cells seeded in 6-well plates were incubated with the diluted DCFH-DA solution at 10 μmol/L for 20 min at 37 °C, followed by the detection using flow cytometry at Ex/Em = 488/525 nm.

### 2.11 RT-qPCR detection

Total RNA in a cell lysate or tissue homogenate was extracted using PureLink™ RNA mini kit (Invitrogen, USA). RNA concentration was calculated according to the OD value read by NanoDrop 2000 at 260 nm and 280 nm. RNA with OD 260/280 > 1.7 was selected for transcription and quantification using the ABI 7500 system (Thermo Fisher, USA). Omniscript RT Kit (QIAGEN, Germany) was used for RNA transcription to cDNA, and QuantiNova SYBR Green PCR Kit (QIAGEN, Germany) for gene quantification, following the product guideline. Finally, gene expression was normalized using 2^−ΔΔCt^ method with the internal reference of *Gapdh*. All primers listed in Table 1 were purchased from Origene (USA).

**Table 1:** Primer sequences used for RT-qPCR.^a^

Gene	Forward primer (5′–3′)	Reverse primer (5′–3′)
*Col2a1*	GCTGGTGAAGAAGGCAAACGAG	CCATCTTGACCTGGGAATCCAC
*Sox9*	CACACGTCAAGCGACCCATGAA	TCTTCTCGCTCTCGTTCAGCAG
*Atp7b*	ATCATCCCAGGACTGTCCGTTC	ATGTTGGCGGACCTGTGTCTCA
*Fdx1*	GTTAGATGCCATTACTGATGAAGAG	CTTCAGGCACACGCACAGTCAT
*Wnt1*	CGAGAGTGCAAATGGCAATTCCG	GATGAACGCTGTTTCTCGGCAG
*Gsk3b*	GAGCCACTGATTACACGTCCAG	CCAACTGATCCACACCACTGTC
*Ctnnb1*	GTTCGCCTTCATTATGGACTGCC	ATAGCACCCTGTTCCCGCAAAG
*Gapdh*	CATCACTGCCACCCAGAAGACTG	ATGCCAGTGAGCTTCCCGTTCAG

a ***Col2a1***: collagen, type II, alpha 1; ***Sox9***: SRY (sex determining region Y)-box 9; ***Atp7b***: ATPase copper transporting beta; ***Fdx1***: ferredoxin 1; ***Wnt1***: wingless-type MMTV integration site family, member 1; ***Gsk3b***: glycogen synthase kinase 3 beta; ***Ctnnb1***: β-catenin; ***Gapdh***: glyceraldehyde-3-phosphate dehydrogenase.

### 2.12 Western blot

Cell lysates or tissue homogenate extracted by RIPA buffer (Beyotime, Shanghai, China) were centrifugated with 12,000 g for 10 min at 4 °C, and then protein in the supernatant was measured by BCA protein concentration detection assay (Beyotime, Shanghai, China). Protein samples were used for SDS-PAGE and subsequently transferred to polyvinylidene fluoride (PVDF) membranes (Millipore, USA). The membranes were incubated with the blocking buffer containing 5 % skim milk for 1 h, followed the incubation with the diluted primary antibodies overnight at 4 °C, including TNF-α (ab183218, 1:1000, Abcam, USA), IL-6 (ab290735, 1:1000, Abcam, USA), MMP13 (ab315267, 1:1000, Abcam, USA), ATP7B (PA5-102826, 1:500, Invitrogen, USA), FDX1 (MA5-50817, 1:500, Invitrogen, USA), WNT1 (ab15251, 1:3000, Abcam, USA), GSK3β (AF5016, 1:2000, affinity, Jiangsu, China), β-catenin (ab224803, 1:400, Abcam, USA) and β-actin (ab213262, 1:1000, Abcam, USA). The membranes were then incubated with anti-rabbit antibody (HRP-linked) (ab6721, 1:5000, Abcam, USA) for 1 h at room temperature. Enhanced chemiluminescence (P0018S, Beyotime, Shanghai, China) was added to the membranes for 1 min. Finally, an X-ray gel imaging system (Bio-Rad, USA) was used for blot imaging. β-actin was used for the internal protein.

### 2.13 Copper measurement

The intracellular copper content was detected by the Cell Copper (Cu) Colorimetric Assay Kit (E-BC-K775-M, Elabscience, Wuhan, China). Tissue homogenate or 200, 000 cells were incubated with 2 mL RIPA buffer for 10 min on the ice, followed by the centrifugation with 12, 000 g for 10 min at 4 °C. Protein concentration in the supernatant was measured by a BCA protein detection kit (E-BC-K318-M, Elabscience, Wuhan, China). Then, 50 μL of the color working fluid was added to the supernatant placed in a 96-well plate at 100 μL per well, at 37 °C for 5-min incubation. OD value read by the microplate at 580 nm was used to calculate intracellular copper content according to the standard curve.

### 2.14 Histopathological staining

**Hematoxylin and eosin staining:** A hematoxylin and eosin kit (C0105S, Beyotime, Shanghai, China) was used to observe the histopathological change in cartilage tissue. After dewaxing and hydration, 4-μm-thick sections of cartilage tissues embedded into paraffin were stained by hematoxylin for 10 min and subsequently stained by eosin for 2 min. Sections were incubated with 70 %, 80 %, 90 % and 100 % ethanol for 10 s one by one, followed by the incubation with xylene for 5 min, twice. Finally, neutral balsam was used for sealing. The histopathological changes were observed using a microscope (Olympus, Japan).

**Safranin-O staining/fast green staining:** A safranin-O staining kit (Solarbio, Beijing, China) was used to investigate the changes in cartilage tissues further. The sections of cartilage tissues, after dewaxing and hydration, were stained with hematoxylin for Weigert staining solution for 5 min and then incubated with safranin-O differentiation fluid for 15 s. After washing, sections were incubated with a fast green staining solution for 5 min, followed by Safranin-O staining solution for 5 min. Sections were incubated with 95 % and 100 % ethanol and xylene for dehydration and transparency. The histological changes in sections sealed in neutral balsam were observed through the microscope. Based on histopathological staining, the Osteoarthritis Research Society International (OARSI) score was evaluated as described in the previous study [27,28].

### 2.15 Immunohistochemical staining

5 μm-thickness sections embedded in paraffin were incubated with 3 % H_2_O_2_ for 10 min at room temperature after dewaxing, hydration and antigen repair. Then, sections were incubated with 5 % goat serum-containing blocking buffer at room temperature for 1 h, followed by the incubation with the diluted anti-FDX1 (dilution: 1:50; 12592-1-AP, Proteintech, Wuhan, China) at 4 °C overnight. Sections were subsequently incubation with secondary antibody (1:1000; SA00004-2, Proteintech, Wuhan, China) for 1 h at 37 °C, and then stained by DAB solution for 5 min. Finally, sections stained by hematoxylin dye were sealed in neutral gum after dehydration and transparency. The expression of FDX1 was observed using a microscope.

### 2.16 Statistical analysis

In the present study, statistical analysis in experimental data is shown as mean ± SD. was performed by SPSS 25.0 software and GraphPad 9.0 software was used for data visualization. Independent *t*-test was used for comparison between two groups, and one-way ANOVA test was used for comparison in ≥3 groups, followed by the post hoc comparison of the Tukey test. All experiments were repeated three times. P < 0.05 indicates the statistical difference.

## 3 Results

### 3.1 Characterization of Cuprorivaite microspheres

The entire preparation process of the microspheres is shown in Fig. 1A. The Cuprorivaite powder was first prepared using the sol-gel method, and then the microspheres were produced using a spray drying process. Our data revealed that the solid content of the powder and the quantity of binder (PVA) are crucial factors influencing the microsphere morphology. Through meticulous optimization, we have established that a 30 % cuprorivaite concentration and a 2 % PVA content yield the most favorable conditions (Fig. S1).

The crystal structure of Egyptian blue, depicted in Fig. 1B, is described in the literature as a calcium and copper sheet silicate. It consists of condensed silicate tetrahedrons arranged in tetragonal arrays forming 4-membered rings, which further generate 8-membered rings, referred to as (SiO)_4_ and (SiO)_8_, respectively. Additionally, the structure includes 5- and 6-membered rings composed of one or two Cu atoms and four Si atoms.

Fig. 1C shows the SEM image of cuprorivaite microspheres, which present a regular spherical structure. EDS analysis (Fig. 1D) detected the presence of Ca, O, Si, and Cu. CaCuSi_4_O_10_ exhibits the characteristic blue color of cuprorivaite, and its XRD pattern displays the typical characteristic peaks consistent with the standard reference card (PDF#12–0512), indicating high crystallinity (Fig. 1E). The particle distribution was shown in Fig. 1F, and the mean size of microspheres was 46 μm. During the 50-day release assay, Cu^2+^ was slowly released from cuprorivaite microspheres, demonstrating their good release properties (Fig. 1G).

### 3.2 Toxicity examination of *in vitro* and *in vivo*

**Fig. 2:**
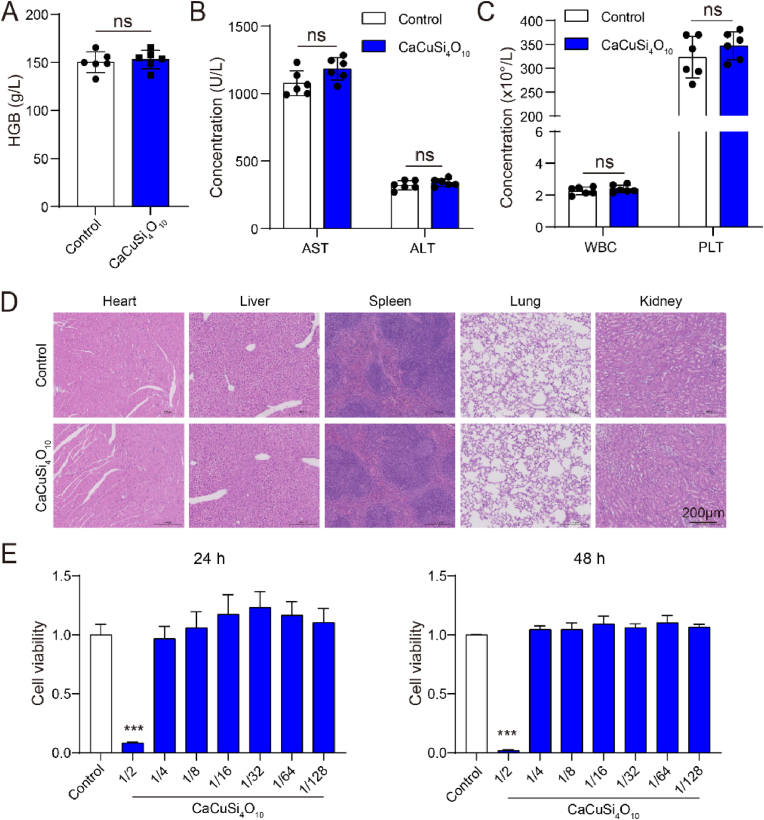
Toxicity examination of *in vitro* and *in vivo*. (A–C) Blood routine tests include hemoglobin (HGB), aspartate aminotransferase (AST), alanine transaminase (ALT), white blood cell (WBC), and platelet (PLT). (D) Hematoxylin and eosin staining for heart, liver, spleen, lung and kidney. (E) Cell counting kit-8 test for cell viability. Compared with the control group, ns indicates not significant, ∗∗∗*P* < 0.001.

To evaluate the security of cuprorivaite microspheres *in vivo*, changes in routine blood indicators and histopathological alterations of mice were investigated. As shown in Fig. 2A–C, there were non-statistical changes in hemoglobin, aspartate aminotransferase, alanine transaminase, white blood cell, and platelet between the control and CaCuSi_4_O_10_ group (*P* > 0.05). Histopathological results indicated that there was no obvious impairment or loss of heart, liver, spleen, lung and kidney tissues in mice receiving CaCuSi_4_O_10_ microspheres as compared to the control group (Fig. 2D). Mouse primary chondrocytes developed no significant change in cell viability for 24 h and 48 h incubation with CaCuSi_4_O_10_ microspheres with dilutions at 1/4-1/128 of the mother extraction solution, and however, CaCuSi_4_O_10_ microspheres with a dilution at 1/2 significantly reduced cell viability after 24 h and 48 h incubation as compared to the control group (*P* < 0.01) (Fig. 2E).

### 3.3 Cuprorivaite microspheres improved inflammatory, cuproptosis and oxidative stress in IL-1β-induced chondrocytes

**Fig. 3:**
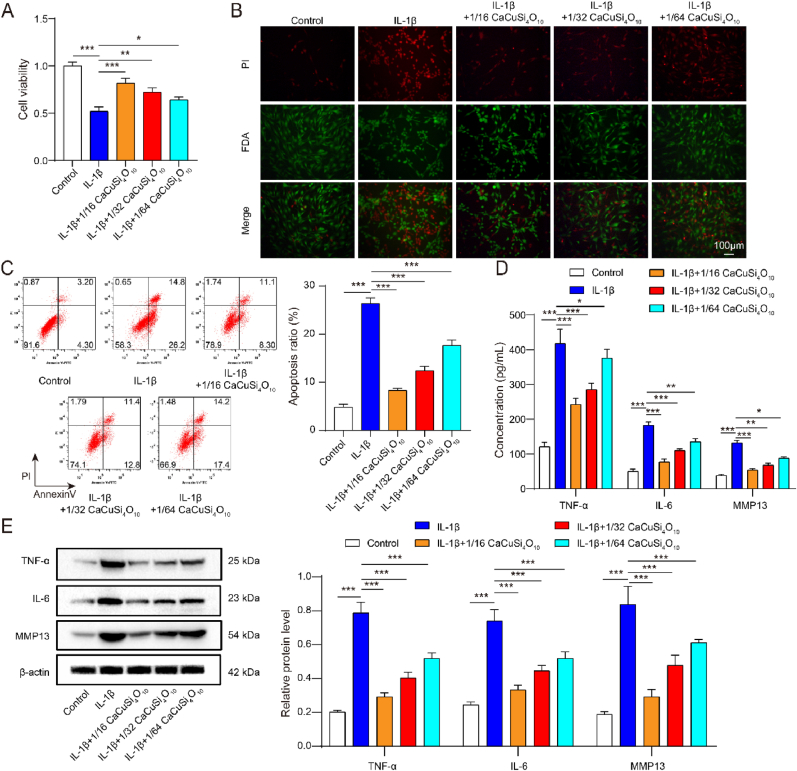
Anti-inflammatory role of Cuprorivaite (CaCuSi_4_O_10_) microspheres in IL-1β-stimulated chondrocytes. (A) Cell counting kit-8 test for cell proliferation. (B) Fluorescein diacetate for cell viability detection. (C) Cell apoptosis by flow cytometry. (D) ELISA detection for TNF-α, IL-6 and MMP13 in cell supernatant. (E) Western blot detection for TNF-α, IL-6 and MMP13 in cell lysates. (F) RT-qPCR quantification for in TNF-α, IL-6 and MMP13. ∗*P* < 0.05, ∗∗*P* < 0.01, ∗∗∗*P* < 0.001.

**Fig. 4:**
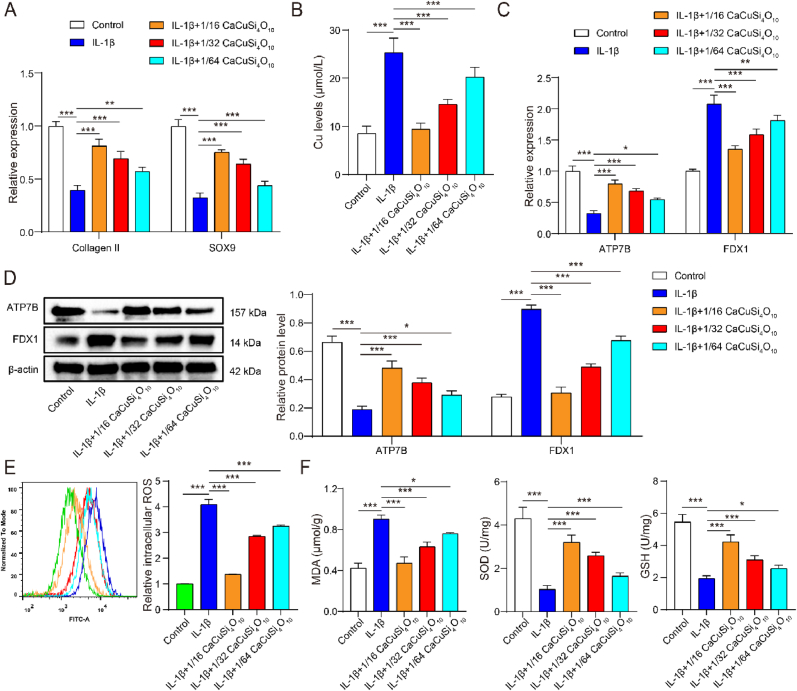
Anti-cuproptosis and anti-oxidative effects of Cuprorivaite (CaCuSi_4_O_10_) microspheres in IL-1β-stimulated chondrocytes. (A) RT-qPCR quantification for extracellular matrix components including collagen II and SOX9. (B) Intracellular copper content. (C) RT-qPCR quantification for cuproptosis biomarkers including ATP7B and FDX1. (D) Western blot detection for ATP7B and FDX1. (E) ROS generation detected by flow cytometry. (F) Oxidative stress detection including MDA, SOD and GSH. ∗*P* < 0.05, ∗∗*P* < 0.01, ∗∗∗*P* < 0.001.

To explore the role of Cuprorivaite (CaCuSi_4_O_10_) in inflammatory chondrocytes, we used Cuprorivaite microspheres with dilutions at 1/16, 1/32, and 1/64 of the mother extraction solution to incubate IL-1β-induced chondrocytes for 24 h. Compared with cell viability in the control group, that in the IL-1β group was significantly decreased; CaCuSi_4_O_10_ microspheres led to the elevation of cell viability in IL-1β-induced chondrocytes, although the promotive role diminished with an increase in dilution factor (*P* < 0.05) (Fig. 3A). According to FDA staining, CaCuSi_4_O_10_ microspheres at all dilutions increased the IL-1β-inhibited live cells (Green in Fig. 3B). Importantly, CaCuSi_4_O_10_ microspheres with a dilution factor at 1/16 had the most promotive effect on chondrocytes, followed by the effects of 1/32 and 1/64 (Fig. 3A and B). As shown in Fig. 3C, the apoptosis in chondrocytes was significantly enhanced after IL-1β stimulation. The IL-1β-induced change in apoptosis was remarkedly reversed by CaCuSi_4_O_10_ microspheres treatment, with the dose-dependent effect that the greater the dilution factor, as shown by the lower the apoptosis (Fig. 3C). In chondrocytes, TNF-α, IL-6 and MMP13 in cell supernatant and cell lysates were increased by IL-1β (*P* < 0.05). CaCuSi_4_O_10_ microspheres caused the reduction in expressions of TNF-α, IL-6 and MMP13 in IL-1β-stimulated chondrocytes following the dilution-dependent effect (Fig. 3D and E). ECM components, including collagen II and SOX9, were inhibited by IL-1β (*P* < 0.05). Expressions of ECM components were reversed by CaCuSi_4_O_10_ microspheres with the dose-dependent effect (*P* < 0.05), including the increased collagen II and SOX9 (Fig. 4A). Also, IL-1β group developed an increased Cu^2+^ concentration as compared to the control group (*P* < 0.05), which was significantly reversed by CaCuSi_4_O_10_ microspheres (*P* < 0.05) (Fig. 4B). CaCuSi_4_O_10_ microspheres playing the dilution-dependent role reduced IL-1β-elevated expressions of FDX1 and inhibited expression of ATP7B (*P* < 0.05) (Fig. 4C and D). IL-1β-induced ROS generation was inhibited by CaCuSi_4_O_10_ microspheres (Fig. 4E). Notably, CaCuSi_4_O_10_ microspheres with a dilution factor at 1/16 had the strongest capability of inhibiting ROS generation, followed by 1/32 and 1/64 (*P* < 0.05) (Fig. 4E). IL-1β evoked oxidative stress in chondrocytes, including an increase in MDA and a decrease in SOD and GSH (*P* < 0.05). CaCuSi_4_O_10_ microspheres had the antioxidant role in IL-1β-stimulated chondrocytes, with the reduction of MDA and the elevation of SOD and GSH (*P* < 0.05) (Fig. 4F).

### 3.4 Cuprorivaite microspheres inhibited Wnt/β-catenin pathway activation in IL-1β-induced chondrocytes

**Fig. 5:**
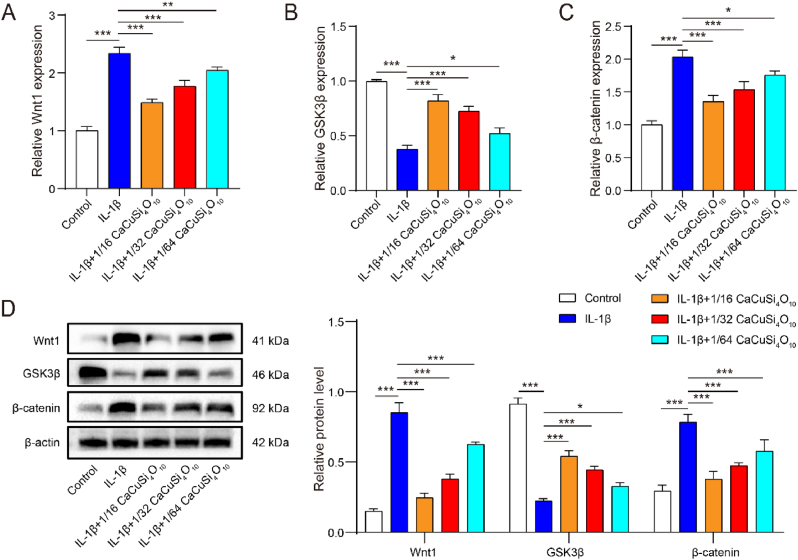
Role of Cuprorivaite (CaCuSi_4_O_10_) microspheres via Wnt/β-catenin pathway in IL-1β-stimulated chondrocytes. (A–C) RT-qPCR quantification for Wnt1 (A), GSK3β (B) and β-catenin (C). (D) Western blot detection for Wnt1, GSK3β and β-catenin in cell lysates. ∗*P* < 0.05, ∗∗*P* < 0.01, ∗∗∗*P* < 0.001.

Then, we investigated the role of CaCuSi_4_O_10_ microspheres in Wnt/β-catenin pathway. As shown in Fig. 5A–D, Wnt1 and β-catenin were significantly increased, and GSK3β developed a significant decrease after IL-1β stimulation. With the dilution-dependent effect, CaCuSi_4_O_10_ microspheres reversed the IL-1β-induced changes in Wnt/β-catenin pathway, with a decrease in Wnt1 and β-catenin and an increase in GSK3β.

### 3.5 Cuprorivaite microspheres improved cuproptosis and oxidative stress via inhibiting Wnt/β-catenin pathway

**Fig. 6:**
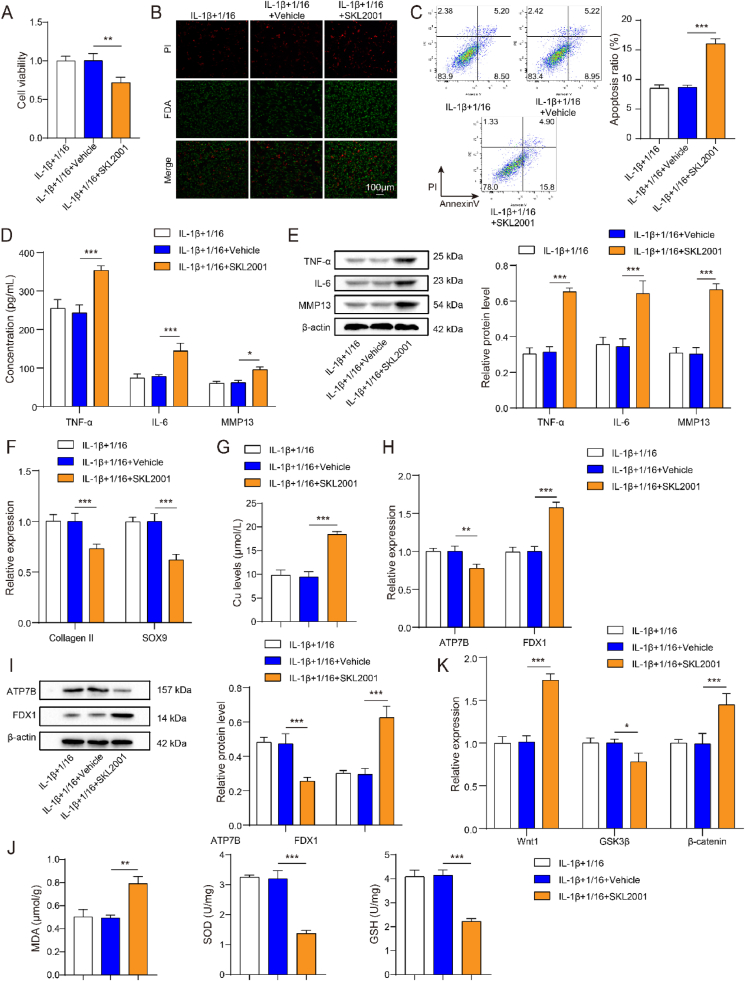
Cuprorivaite (CaCuSi_4_O_10_) microspheres improved IL-1β-induced injury in chondrocytes via inhibiting Wnt/β-catenin pathway. (A) Cell counting kit-8 test for cell viability. (B) Fluorescein diacetate for cell viability detection. (C) Cell apoptosis by flow cytometry. (D) ELISA detection for TNF-α, IL-6 and MMP13 in cell supernatant. (E) Western blot detection for TNF-α, IL-6 and MMP13 in cell lysates. (F) RT-qPCR quantification for extracellular matrix components including collagen II and SOX9. (G) Intracellular copper content. (H) RT-qPCR quantification for cuproptosis biomarkers including ATP7B and FDX1. (I) Western blot detection for ATP7B and FDX1. (J) Oxidative stress detection including MDA, SOD and GSH. (K) RT-qPCR quantification for Wnt1, GSK3β and β-catenin. ∗*P* < 0.05, ∗∗*P* < 0.01, ∗∗∗*P* < 0.001.

To determine whether Cuprorivaite modulates Wnt/β-catenin pathway in OA, SKL2001 was used to activate Wnt/β-catenin pathway in cells. As shown in Fig. 6A, in IL-1β-stimulated chondrocytes, SKL2001 significantly inhibited microsphere-increased cell viability (*P* < 0.05). FDA staining assay also validated SKL2001 could reverse the role of CaCuSi_4_O_10_ microspheres in IL-1β-stimulated chondrocytes (Fig. 6B). SKL2001 resulted in an increase in cell apoptosis in chondrocytes undergoing CaCuSi_4_O_10_ microspheres treatment (*P* < 0.05) (Fig. 6C). SKL2001 stimulated the pro-inflammatory in chondrocytes, with increases in TNF-α, IL-6 and MMP13 (*P* < 0.05) (Fig. 6D and E). ECM components were changed after SKL2001, including the decreases in Collagen II and SOX9 as compared to CaCuSi_4_O_10_ microspheres treatment (*P* < 0.05) (Fig. 6F). SKL2001 caused the increases in intracellular copper and FDX1 and the decrease in ATP7B (*P* < 0.05) (Fig. 6G–I). SKL2001 played the pro-oxidant role in chondrocytes undergoing CaCuSi_4_O_10_ microspheres treatment. As shown in Fig. 6J, there were the decreases in SOD and GSH and the increase in MDA after SKL2001 (*P* < 0.05). The microsphere-induced changes in Wnt/β-catenin pathway were reversed by SKL2001, including the elevated Wnt1 and β-catenin and the reduced GSK3β (*P* < 0.05) (Fig. 6K).

### 3.6 Cuprorivaite microspheres improved cuproptosis and oxidative stress in OA mice via inhibiting Wnt/β-catenin pathway

**Fig. 7:**
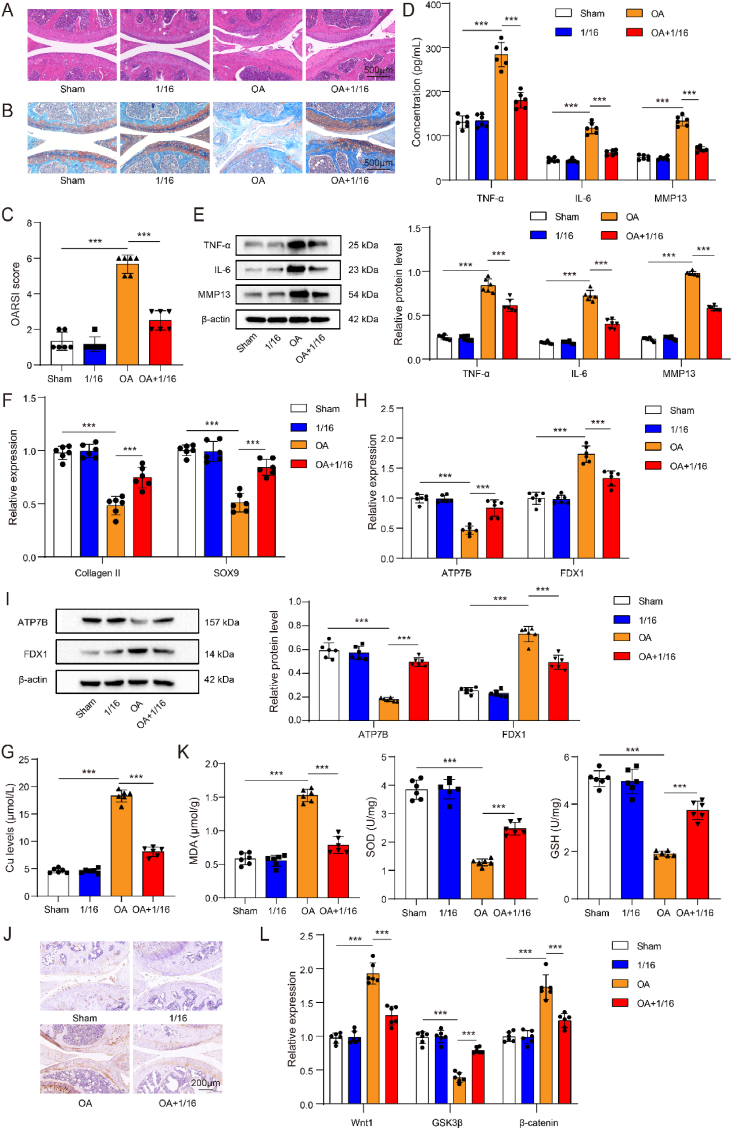
Validation of mechanism of Cuprorivaite (CaCuSi_4_O_10_) microspheres in OA. (A) Hematoxylin and eosin staining. (B) Safranin-O staining/fast green staining. (C) OARSI score. (D) ELISA detection for TNF-α, IL-6 and MMP13 in the serum. (E) Western blot detection for TNF-α, IL-6 and MMP13 in cartilage tissue. (F) RT-qPCR quantification for extracellular matrix components including collagen II and SOX9 in cartilage tissue. (G) Copper content in cartilage tissue. (H) RT-qPCR quantification for cuproptosis biomarkers including ATP7B and FDX1 in cartilage tissue. (I) Western blot detection for ATP7B and FDX1 in cartilage tissue. (J) Representative images of FDX1 expression in cartilage tissue detected by immunohistochemistry. (K) Oxidative stress detection including MDA, SOD and GSH in cartilage tissue. (L) RT-qPCR quantification for Wnt1, GSK3β and β-catenin in cartilage tissue. ∗∗∗*P* < 0.001.

**Fig. 8:**
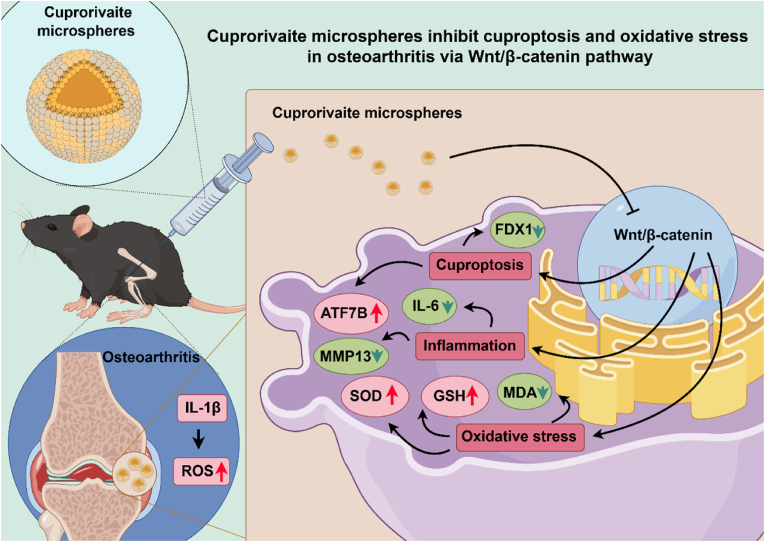
The potential mechanism by which Cuprorivaite microspheres improved cartilage injury in OA. By Figdraw.

A mouse model of OA was used to validate the protective mechanism of CaCuSi_4_O_10_ microspheres. As compared to sham group, the OA group exhibited reduced cartilage thickness and incomplete cartilage layers, with tissue degradation; however, the injury was improved by the administration of CaCuSi_4_O_10_ microspheres (Fig. 7A). Safranin-O/fast green staining presented the reduction in cartilaginous matrix in mice with OA, and CaCuSi_4_O_10_ microsphere promoted cartilaginous matrix close to mice in the sham group (Fig. 7B). Fig. 7C suggests that mice in the OA group showed an increased OARSI score as compared to those in the sham group (*P* < 0.05); OARSI in the OA+1/16 group had lower OARSI than that in OA group (*P* < 0.05). Compared to the sham group, the OA group developed the enhancement of TNF-α, IL-6 and MMP13 in the serum and cartilage tissues (*P* < 0.05); CaCuSi_4_O_10_ microspheres significantly inhibited these pro-inflammatory factors (*P* < 0.05) (Fig. 7D and E). OA led to cartilage degradation in mice, as evidenced by the decreases in SOX9 and collagen II (*P* < 0.05), but CaCuSi_4_O_10_ microspheres improved the injury in cartilage (*P* < 0.05) (Fig. 7F). Importantly, CaCuSi_4_O_10_ microspheres decreased OA-increased copper content in the serum (*P* < 0.05) (Fig. 7G). Also, CaCuSi_4_O_10_ microspheres lead to the upregulated ATP7B and the downregulated FDX1 in mice with OA (*P* < 0.05) (Fig. 7H–J). There was obvious oxidative stress in mice with OA as compared to mice in the sham group, including the reduction in SOD and GSH and the elevation in MDA (*P* < 0.05); CaCuSi_4_O_10_ microspheres could significantly reverse the OA-induced oxidative stress (*P* < 0.05) (Fig. 7K). Wnt/β-catenin pathway was altered in mice with OA, including the elevation in Wnt1and β-catenin and the reduction in GSK3β (*P* < 0.05), which was reversed by CaCuSi_4_O_10_ microspheres (*P* < 0.05) (Fig. 7L). Collectively, we demonstrated that CaCuSi_4_O_10_ microspheres could inhibit cuproptosis and oxidant injury in the cartilage via causing Wnt/β-catenin pathway inactivation during OA (Fig. 8).

## 4 Discussion

Cuproptosis and oxidative stress are two major factors that affect OA progression [11,29]. Copper-based materials are superior modulators for scavenging oxidative stress [30], which may also influence cuproptosis due to the relationship between copper metabolism and oxidative stress in mitochondria [10]. In this study, we synthesized Cuprorivaite microspheres and demonstrated that these microspheres inhibit cuproptosis and oxidative stress in IL-1β-treated chondrocytes and a mouse OA model. The underlying molecular mechanism may involve the suppressive effect of Cuprorivaite microspheres on the Wnt/β-catenin pathway.

OA is accompanied by an excessive inflammatory response and oxidative stress, as evidenced by the massive release of pro-inflammatory factors, overproduction of ROS, and downregulation of antioxidant enzymes and antioxidants [31,32]. Our results demonstrate that IL-1β significantly inhibited cell viability and promoted apoptosis, indicating that IL-1β stimulation can lead to apoptosis in chondrocytes. With the application of Cuprorivaite microspheres, IL-1β stimulated chondrocytes exhibited a reduction in pro-inflammatory cytokines and ROS generation. More importantly, Cuprorivaite microspheres also mitigated oxidative stress and counteracted IL-1β induced apoptosis, suggesting that Cuprorivaite microspheres have the potential to reverse IL-1β evoked damage in chondrocytes. It is widely acknowledged that OA develops ECM degradation in cartilaginous tissue due to MMPs [32]. In these IL-1β-stimulated chondrocytes, we found a reduction in ECM components including collagen II and an elevation in MMP13, which is consistent with previous findings [33]. However, Cuprorivaite microspheres reversed the ECM degradation in IL-1β-stimulated chondrocytes. Intriguingly, the anti-inflammatory and antioxidant effects of Cuprorivaite microspheres on cartilage were also observed in our mouse OA model. Therefore, our study first demonstrated the therapeutic effect of Cuprorivaite microspheres on OA both *in vitro* and *in vivo*.

Cuproptosis is a specific non-apoptotic programmed death type as compared to other death mechanisms such as apoptosis, pyroptosis and necroptosis [8]. Modulation of cuproptosis during OA may exert therapeutic effects. Research has demonstrated that bioactive Cu^2+^ shows an anti-inflammation role in chondrocytes and drives cartilage regeneration in OA [34]. Our study generated Cu^2^^+^-releasing Cuprorivaite microspheres for OA therapy to achieve this goal. A series of cuproptosis-related genes present differential expression in patients with OA as compared to normal controls, such as FDX1 [12,35]. In the mechanism of cuproptosis, a downregulation in ATP7B that mediates the extracellular release of Cu^+^ and upregulation in FDX1 promoting Cu^2+^ reduced to toxic Cu^+ 10^. In the present study, we noticed IL-1β induced cuproptosis in chondrocytes for the first time, including a decrease in ATP7B and increases in FDX1 and cellular Cu^2+^, suggesting cuproptosis was activated in OA. Intriguingly, we further observed Cuprorivaite microspheres could inhibit cuproptosis in IL-1β-stimulated chondrocytes, as evidenced by ATP7B upregulation and the reduction in FDX1 and cellular Cu^2+^. The mechanism by which Cuprorivaite microspheres repressed cuproptosis may involve GSH. Cu^2+^ directly entering cells is reduced to toxic Cu^+^ with the catalyzation of FDX1, thereby leading to the lipoylated DLAT that drives the lipoylation of mitochondrial protein to trigger cuproptosis [8]. GSH can chelate Cu^+^ to interrupt the interaction between DLAT and Cu^+10^. We have demonstrated Cuprorivaite microspheres, which resulted in the elevation of GSH in chondrocytes. Thus, the elevated GSH may bind to Cu^+^ and subsequently block the Cu^+^-related mechanism in cuproptosis, which is merely a hypothesis requiring further verification. Moreover, the controlled release of Cu^2+^ ions from Cuprorivaite microspheres could exert a dampening effect on the IL-1β-induced activation of chondrocytes, potentially leading to a negative feedback mechanism. This phenomenon is consistent with observations in the murine OA model. Copper disorder may contribute to the dysfunction of bone metabolism [36]. Cuprorivaite microspheres may also modulate the copper homeostasis of subchondral bone in OA, which further investigations are needed. In essence, Cuprorivaite microspheres appear to have the capacity to suppress cuproptosis in OA.

Wnt/β-catenin pathway is the key signaling pathway involved in physiological cellular processes such as cell proliferation, apoptosis and differentiation [18]. β-catenin activated by Wnt-dependent cascade plays the role of elevating the transcription of Wnt-responding genes, as the coactivator of TCF/LEF family transcription factor [18]. The abnormally activated Wnt/β-catenin pathway has been demonstrated to facilitate the occurrence and development of OA. For example, Li et al. concluded that Wnt/β-catenin pathway activation caused the OA-like phenotype in the *in vitro* and *in vivo* model, which was reversed by the inhibitor of Wnt/β-catenin pathway [23]. In our study, a decrease in GSK3β and the increases in Wnt1 and β-catenin were present in IL-1β stimulated chondrocytes and OA mice, suggesting Wnt/β-catenin pathway was elevated in OA. Importantly, we further observed that Cuprorivaite microspheres could inhibit the abnormal activation of Wnt/β-catenin pathway, and the agonist of Wnt/β-catenin pathway obviously offset the protection role of Cuprorivaite microspheres in IL-1β-stimulated chondrocytes, indicating Wnt/β-catenin pathway may be the significant therapeutic target of Cuprorivaite microspheres in OA. Moreover, we found that the Wnt/β-catenin pathway might also mediate the inhibition of Cuprorivaite microspheres to cuproptosis since the activator of Wnt/β-catenin pathway resulted in the accumulation of Cu^2+^, increase in FDX1, and decrease in ATP7B. This appears to be the first time that there is a relationship between the Wnt/β-catenin pathway and cuproptosis in OA. β-catenin is the key factor of transcription activation [37]. Thus, β-catenin, on the one hand, has the potential to participate in the expression of cuproptosis-related genes at the transcriptional level. On the other hand, β-catenin plays the role of maintaining GSH homeostasis via modulating the transcription process of genes related to the GSH-metabolic cascade [38]. Indeed, the Cuprorivaite microspheres effectively inhibit the activation of the Wnt/β-catenin signaling pathway in OA, thereby mitigating oxidative stress. This is evidenced by the upregulation of GSH, which in turn chelates Cu^+^, leading to a reduction in cuproptosis. Such reduction is manifested by an increase in FDX1 expression and a decrease in ATP7B levels (Fig. 8).

In the present study, we established the Cuprorivaite microspheres and investigated their cartilage protection in OA progression. To our knowledge, this is the first study reporting that these microspheres contribute to the treatment of OA. However, there were some limitations in the present investigation. We were unable to investigate the mechanism by which Cuprorivaite microspheres exert their effects on cuproptosis, as well as to delineate the precise relationship between extracellular and intracellular copper ions. The alterations in cuproptosis factors related to the Wnt/β-catenin pathway in response to Cuprorivaite microspheres in the context of OA remain unresolved. In our subsequent studies, we plan to employ sequencing technology and bioinformatics analysis to identify differentially expressed genes and proteins in OA models with and without Cuprorivaite treatment. Additionally, we are currently exploring advanced nanotechnological approaches to precisely modulate the concentration of copper ions within Cuprorivaite microspheres as part of our ongoing modifications.

## 5 Conclusion

In this work, we constructed the Copper-based bioactive microspheres consisting of Cuprorivaite, and demonstrated its protection in chondrocyte survival during OA via inhibiting inflammation, oxidative stress and cuproptosis. Also, we demonstrated Wnt/β-catenin pathway might mediate the protective mechanism of Cuprorivaite microspheres in OA. Our findings provide a novel insight into the relationship between OA and cuproptosis, and reveal the potential role of Cuprorivaite in cuproptosis.

## Declaration of generative AI and AI-assisted technologies in the writing process

During the preparation of this work the author(s) used ChatGPT-4o in order to linguistic amendment. After using this tool/service, the author(s) reviewed and edited the content as needed and take(s) full responsibility for the content of the publication.

## CRediT authorship contribution statement

**Bo Li:** Writing – original draft, Methodology, Investigation, Formal analysis, Data curation. **Tongmeng Jiang:** Writing – review & editing, Supervision, Funding acquisition, Conceptualization. **Juan Wang:** Methodology, Investigation. **Hongping Ge:** Resources, Methodology. **Yaqi Zhang:** Data curation. **Tong Li:** Validation. **Chen Wang:** Writing – review & editing, Supervision, Funding acquisition, Conceptualization. **Weiguo Wang:** Writing – review & editing, Supervision, Conceptualization.

## Declaration of competing interest

The authors declare that they have no known competing financial interests or personal relationships that could have appeared to influence the work reported in this paper.

## Data Availability

Data will be made available on request.
