# Supplementary material for: Cuprorivaite microspheres inhibit cuproptosis and oxidative stress in osteoarthritis via Wnt/β-catenin pathway
Source: Mater Today Bio. 2024 Oct 16;29:101300. doi: 10.1016/j.mtbio.2024.101300 (PMC11513804; doi:10.1016/j.mtbio.2024.101300)
Supplement: Multimedia component 1 [file mmc1.docx]

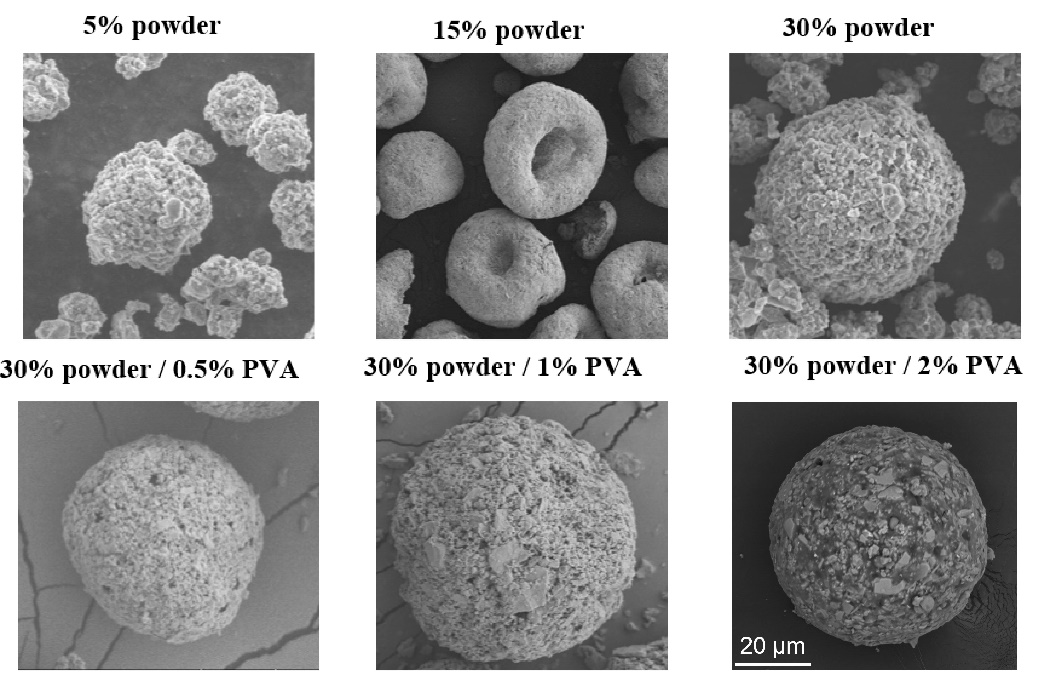


Figure S1. The effect of different powder solid contents and PVA addition amounts on microsphere morphology.
